# Inductive heating and flow chemistry – a perfect synergy of emerging enabling technologies

**DOI:** 10.3762/bjoc.18.70

**Published:** 2022-06-20

**Authors:** Conrad Kuhwald, Sibel Türkhan, Andreas Kirschning

**Affiliations:** 1 Institute of Organic Chemistry, Leibniz University Hannover, Schneiderberg 1b, 30167 Hannover, Germany

**Keywords:** catalysis, enabling technologies, flow chemistry, inductive heating, multistep synthesis, nanoparticles

## Abstract

Inductive heating has developed into a powerful and rapid indirect heating technique used in various fields of chemistry, but also in medicine. Traditionally, inductive heating is used in industry, e.g., for heating large metallic objects including bending, bonding, and welding pipes. In addition, inductive heating has emerged as a partner for flow chemistry, both of which are enabling technologies for organic synthesis. This report reviews the combination of flow chemistry and inductive heating in industrial settings as well as academic research and demonstrates that the two technologies ideally complement each other.

## Introduction

**Figure 1:**
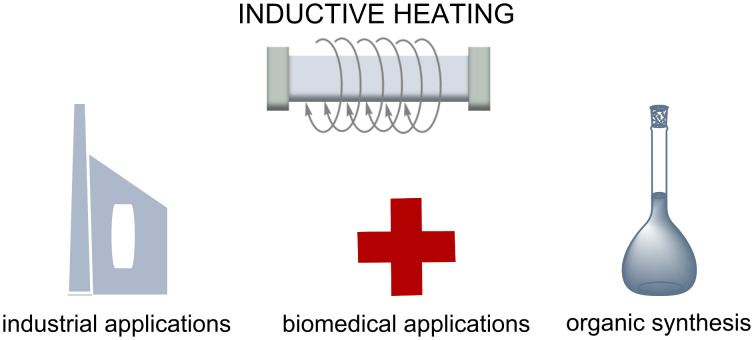
Inductive heating, a powerful tool in industry and the Life Sciences.

Several decades ago, inductive heating was introduced as an indirect technique in various applications, including industrial manufacturing, synthetic chemistry [1–3], and medicine (Figure 1) [4–7]. Compared to microwave heating [8–9], the other major indirect heating technique, inductive heating has several advantages. Technically, the system is composed of an inductive coil and an alternating current (AC) generator. The material to be heated is usually located in the interior of the coil or in its vicinity, so that the heat is not generated by convection across a surface. Compared to heating under microwave irradiation, the system does not need to be encased for safety reasons. Inductive heating of materials is extremely fast with the best determined power transfer value of all heating technologies [10]. It has therefore found wide industrial application for heating large metallic objects and workpieces. It is used for bending tubes, bonding, welding, sintering, and annealing of metals and alloys [11]. In addition to steel and alloys, glass or silicon are also heated or melted under inductive heating conditions [12]. In the last decade, inductive heating has also been used for bonding, heating rubber, deforming plastic, or shrinking workpieces [13–15]. These materials are not conductive like steel, copper, or alloys, so another mechanism must take hold to introduce heat. This is often achieved by embedding small superparamagnetic ferromagnetic or ferrimagnetic nanoparticles into these materials. For this purpose, superparamagnetic iron oxide nanoparticles (SPION) are most commonly used, of which the main forms are magnetite (Fe_3_O_4_) and its oxidized form maghemite (γ-Fe_2_O_3_). Although cobalt and nickel are also highly magnetic materials, they are less common due to their inherent toxicity and ease of oxidation.

Another branch of research involving SPIONs focuses on developments in nanoscience, nanomedicine, and nanoparticle-assisted imaging, diagnosis, and drug delivery [4–7], an area that is not covered in this report.

## Review

### 1 Theoretical background of electromagnetic induction

To better understand the mechanisms of inductive heating, some basic physical principles are first explained. This type of heating depends on various structural, morphological, chemical, and physical properties of the materials to be heated. When a suitable receiver is placed in an alternating electromagnetic field, this energy is converted into heat, apart from minor losses due to convection, conduction, and thermal radiation. This conversion of energy into heat takes place according to three different principles, which depend on the properties of the material.

#### 1.1 Hysteresis loop

**Figure 2:**
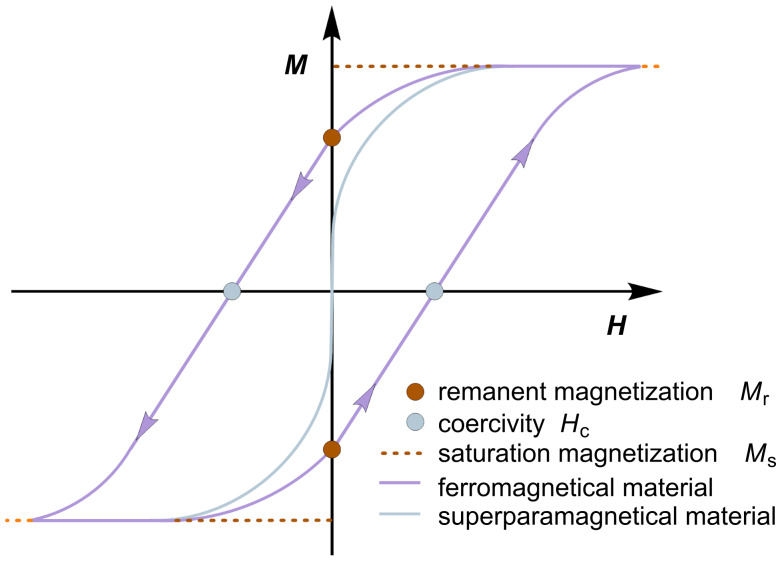
Electric displacement field of a ferromagnetic and superparamagnetic material.

The orbital motion and electron spin profile of a material determine its magnetic properties. Ferromagnetic (FM) materials have unpaired electron spins that couple in space and provide a strong magnetic force. However, ferromagnetic materials consist of multiple domains. In a magnetic field, electron spins align within a domain, but commonly not in all domains. As such, ferromagnetic materials consume the energy to grow domains in the direction of the field. However, the multidomain state becomes energetically unfavorable once the material under consideration reaches a certain size such that the energy required to form a domain wall is higher than the energy required to maintain the magnetostatic energy of a single domain. In ferromagnetic single-domain materials, the spins align in the same direction and act as a giant magnetic moment [16–17]. The coupling of these spins to the crystal lattice is called hysteresis. When a magnetic field is applied, the electromagnetic energy of the atoms is transferred to the lattice in the form of heat, which is undesirable for many applications of magnetic materials. This process is therefore referred to as magnetic loss. The amount of energy loss per cycle of magnetic field generation is interpreted as the magnetization of a material in a hysteresis loop, which is defined as magnetic hysteresis loss (Figure 2). It is characterized by three parameters: 1) the saturation magnetization (*M*_s_), at which the material reaches its maximum in the magnetic field, 2) the remanent magnetization (*M*_r_), which is retained by the material when the magnetic field is removed, and 3) the coercivity (*H*_c_), which is the magnetic field required to demagnetize the sample and determines the heat release to the surrounding media. These three parameters are critical to the heat release of magnetic nanoparticles and can vary for different particle types. Coercivity is an inherent property of magnetic nanoparticles that reaches a maximum at a critical diameter of magnetic nanoparticles from multidomain to single domain structures. In hysteresis curves, the area between *M*_r_ and *H*_c_ is correlated with the energy absorbed per mass. For ferromagnetic materials, the area indicates that their magnetic heating mechanism depends on hysteresis losses.

#### 1.2 Néel relaxation

Magnetic particles consisting of a single domain have a remanent magnetization (*M*_r_) of zero; therefore, they lack the hysteresis contribution in the heating process (Figure 2). Their mechanism of electromagnetic energy dissipation is described by the Néel relaxation mechanism [18]. This phenomenon is called superparamagnetism (SPM) and occurs with decreasing particle size when reaching the nanoscale. The energy barrier of such superparamagnetic nanoparticles to reverse magnetization is directly related to magnetic anisotropy and particle volume [19–20]. In external magnetic fields, these spins rotate in the direction of the magnetic field direction and the axis of magnetic moment fluctuates along the magnetocrystalline anisotropy axis. Néel has described the relationship between the relaxation time of the thermal fluctuations of the magnetic moments of the individual domains and the uniaxial anisotropy. In Néel relaxation, the energy barrier for the remanence of magnetization decreases with smaller particle volume. The Néel relaxation process can be observed in dry, powdered single-domain nanoparticles or in immobilized nanoparticles, e.g., when embedded in tumor tissue.

#### 1.3 Joule effect

Eddy currents or Foucault currents are generated by an oscillating electromagnetic field that penetrates the resistance of a magnetically conducting receiver and releases energy through the Joule effect [21–22]. The heating power in eddy currents is directly correlated with the square of the applied frequency and field amplitude. In contrast to the two previous effects, the distribution of current density is not homogeneous when a conductor is introduced into an oscillating electric current. It decreases exponentially starting from the surface with increasing distance, e.g., into the depth of the material. The fact that the heat is not evenly distributed, but is mainly located on the surface, is called the skin-depth effect. It should be noted that this effect decreases significantly with increasing frequency. Two further parameters to be considered for inductively heated materials are the Curie temperature (TC) and the blocking temperature (TB). They mark the phase transition from ferromagnetic to paramagnetic and from ferromagnetic to superparamagnetic materials, respectively. These values represent the thermal limit up to which the materials can be inductively heated, since above this point they lose their permanent magnetism [23].

### 2 Inductive heating in industrial applications under flow conditions

#### 2.1 General remarks

**Figure 3:**
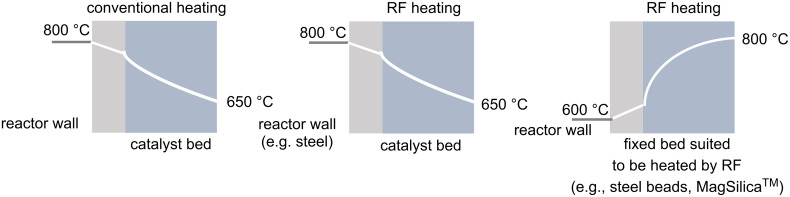
Temperature profiles of reactors heated conventionally and by RF heating (Figure 3 redrawn from [24]).

Energy efficiency is one of the most important cost factors in industrial processes, especially for high temperature reactions in fixed-bed reactors. In general, conductive particles or (superpara)magnetic nanoparticles are suitable as fixed-bed materials for heat generation by applying an external oscillating electromagnetic field. Due to the high specific surface area of these bulk materials, rapid heat transfer by radio frequency (RF) heating is possible (Figure 3). RF-induced heating offers several advantages for use in high-temperature reactions. Advantageously, the heat is generated directly within the reactors, bypassing the problem of thermal gradients. Another advantage of generating heat directly at or near the catalyst surface arises from the potential for hot spots to form, which can substantially exceed the volume temperature of the surrounding reaction medium and lead to significant accelerations of chemical reactions. It is also important that the reactor wall is not exposed to the high temperatures in this process, which has safety implications. Finally, the desired temperature is reached more quickly compared to convective heating and better temperature control can be ensured, e.g., by IR pyrometers.

#### 2.2 Application of trickle bed reactor systems for isopulegol production

Berenguer-Murcia et al. [25] developed a near isothermal micro trickle bed reactor operated by radiofrequency (RF; 300 kHz) heating of nickel ferrite particles (110 µm) deposited in the fixed bed. To achieve near-isothermal conditions at a reactor length of 50 mm, at least three heating zones were set up. The fixed bed was composed of alternating catalyst and heating zones. The heating zones consisted of a mixture of nickel ferrite particles and glass spheres with a particle size of 110 µm. Conventionally heated trickle bed reactors using externally located heating devices suffer from uneven temperature distribution in the reactor bed and the formation of hot spots that can lead to rapid deactivation of the catalyst.

**Scheme 1:**
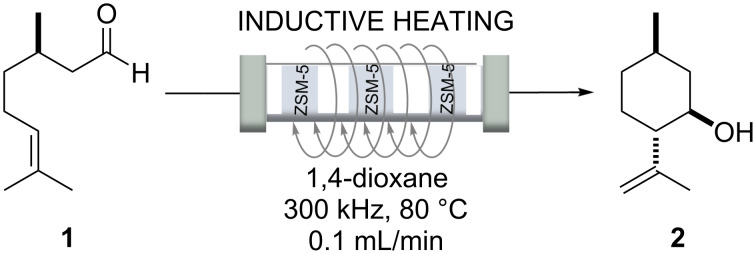
Continuous flow synthesis of isopulegol (**2**) from citronellal (**1**).

The authors selected the synthesis of isopulegol (**2**) from citronellal (**1**) as test reaction (Scheme 1). Thus, citronellal (**1**) was cyclized to isopulegol in the heating zone. This was achieved at 80 °C in 1,4-dioxane using a Zeolite-encapsulated magnetic nickel ferrite nanoparticles (NiFe_2_O_4_@TiO_2_@ZSM-5) catalyst, an aluminosilicate zeolite, which gave best results due to its high Brønsted acidity [26]. Using inductive heating resulted in a highly improved catalytic system that showed long-term stability. This example is of relevance for the fragrance and flavour industries, as isopulegol (**2**) can be transformed into menthol in one step by catalytic hydrogenation.

#### 2.3 Dry and steam methane reforming

**Scheme 2:**
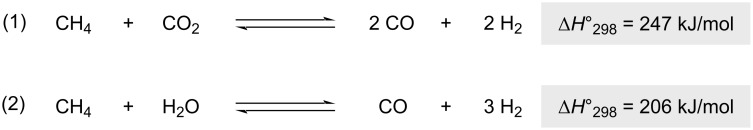
Dry (reaction 1) and steam (reaction 2) methane reforming.

The commencement of the energy transformation is associated with the search for alternative and more environmentally friendly energy sources [27]. The dry reforming of methane is a particularly interesting process in this context (Scheme 2, reaction 1). A second variant is the steam methane reforming process. Since complex solids such as wood, sewage sludge or municipal waste cannot be evaporated, they are reformed using supercritical water on a heterogeneous catalyst at 250–300 bar, 400–550 °C, and a large excess of water [28]. The former process is the preferred route for large-scale production of syngas from biogas [29], while the latter is the main catalytic route [30].

The intrinsic problem with these processes is the extremely high temperature required, typically above 700 °C (ambient pressure) [27]. Since most such processes do not operate at ambient pressure, much higher temperatures around 950 °C are actually required. At these high temperatures, the selectivity of the process is a challenge. Possible side reactions such as hydrogenation of CO and CO_2_, decomposition of CH_4_, and the Boudouard reaction lead to the formation of elemental carbon [31]. Another problem is the Curie temperature (*T*_C_) associated with the material. Depending on the composition of the stainless steel, this is around 750 °C [32]. In 2017, Mortensen's team performed the reaction for the first time with inductively heated Ni-Co NP alloys deposited on a magnesium aluminate (MgAl_2_O_4_) spinel [33]. The alloy prepared specifically for this case contained 12.6 wt % Ni and 9.0 wt % Co with a Curie temperature above 800 °C. By using a high Co content (*T*_C_ = 1115 °C), they were able to maintain ferromagnetic properties even at very high temperatures. Since the addition of nickel further catalyzes this reaction, complete conversions with low carbon formation could be obtained at low flow rates. At higher flow rates, reaction kinetics was the limiting factor. Later, it was found that by doping the alloy with small amounts of copper, almost complete conversion (95%) could be achieved at lower electromagnetic fields and higher flow rates (Q = 152 NL/h) [34]. Not only was less carbon formation observed with this new material, but also little to no reduction in catalytic activity. Although this is not yet the most efficient process that could be used on a large scale, it is a remarkable application for inductive heating.

#### 2.4 CO_2_ storage and release under RF heating

**Scheme 3:**
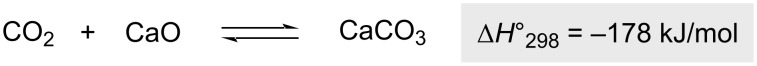
Calcination and RF heating.

The climatic changes are associated, among other factors, with increasing emissions of CO_2_ into the atmosphere. The group of Rebrov et al. investigated the storage of CO_2_ in CaO via the calcination process using inductive heating (Scheme 3) [35].

The cycle can be divided into two individual processes: First, the carbonation step, in which the CaO absorbs the CO_2_ to form the calcium carbonate at 650–680 °C, and second, the subsequent calcination step, in which the CaO-based sorbent is sintered at temperatures of 850–950 °C to release the captured CO_2_ and form CaO again. The inductive method is a very easy to implement and cost-effective system that can be installed in production plants. A higher desorption rate (15.4%) and a lower degree of sintering of the sorbent were observed with the IH method compared to conventional heating methods, coupled with shorter cycle and start-up times. Rebrov et al. also suggested that the system can be used during periods of low power consumption to reduce the load on the electrical system.

#### 2.5 Preparation of hydrocarbons (the Fischer–Tropsch process)

Monodisperse Fe@FeCo core shell nanoparticles as well as Fe(0) nanoparticles with a Ru(0) layer exhibit large heating capability when exposed to an external oscillating electromagnetic field. These particles combine magnetic and surface catalytic properties and thus have been employed in the Fischer–Tropsch process. The heating performance is characterized by the specific absorption rate (SAR) of the material. The tested materials showed high SAR values when brought into an electromagnetic field of 50 mT at a frequency of 54 kHz. Under these conditions the particles were able to catalyze the hydrogenation of CO. The presence of ruthenium increased the catalytic activity and allowed the catalytic process to be carried out at lower reaction temperatures, which was explained by the fact that the surface temperature of the nanoparticles was in fact significantly higher than 200 °C. It was also not necessary to implement an additional heating device for the outer reactor wall. Thus, this process represents a promising example of "cold magnetic catalysis" as it is termed in the paper [36].

#### 2.6 Methane production (“Sabatier” process)

One of the largest challenges for sustainable power generation is to create a system in which all energy sources can be converted into each other efficiently and according to demand. In the so-called "power to gas" (PtG) technology, an efficient catalytic approach has been missing to date [37–38]. A very challenging catalytic process, which is gaining importance in this context, is the Sabatier reaction.

**Scheme 4:**
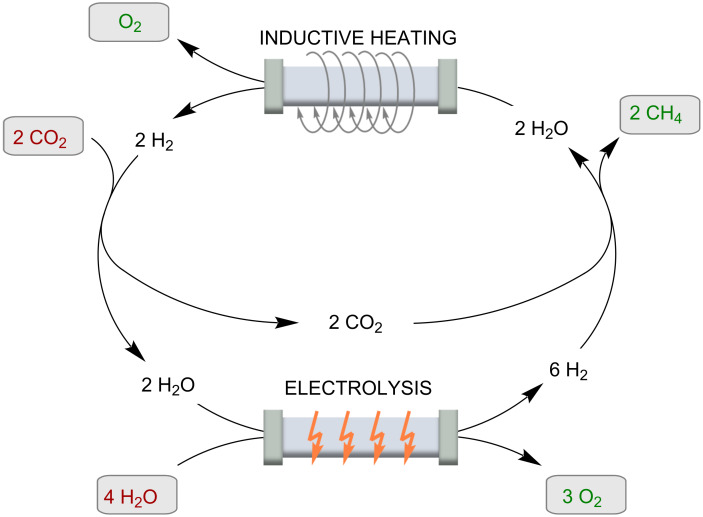
The continuously operated “Sabatier” process.

In this reaction, CO_2_ and H_2_ are converted to CH_4_ (Scheme 4). This process can be used as a catalytic cycle in combination with electrolysis of water to produce the hydrogen exactly when the demand requires it. The reactions are very powerful and can represent an important access to synthetic fuels. This is important not only in the context of energy storage, CO_2_ reduction, and climate change prevention, but also because they provide a country-independent source of energy. However, for use in a continuous flow system, the catalyst must have extremely high SAR and catalytic activity. Recently, a series of promising systems have been produced for this purpose. These include (Fe_2.2_C) NPs [39], (ICNPs@Ni; 29 wt % Ni), and (ICNPs@Ru 1 wt % Ru) on a silica-alumina support (SiRAlox). The Ru nanoparticles far outperformed the previously known catalysts. A methane yield of 93% with complete selectivity could be achieved with high flow rates (125 mL/min; reactor dimensions according to SI: 2 cm diameter and 1 cm height of catalyst filling) in an external electromagnetic field of 28 mT. Heating can be problematic when exothermic reactions are performed. To prevent catalyst deactivation, the group of Giambastiani et al. developed homogeneously sized Ni nanoparticles (4 ± 1 nm) decorated on an oxidized carbon felt (OCF) matrix [40]. A laser pyrometer was used to measure the temperature of the catalyst bed in the quartz reactor. The inductor was controlled by a proportional-integral-derivative (PID) controller, which regulates the temperature. This feedback loop allowed the temperature of the catalyst bed to be fine-tuned and adjusted in real time to suit the conditions.

#### 2.7 Biofuel production

The increasing demand for renewable energy sources goes hand in hand with the sustainable and efficient use of naturally occurring waste. Two factors that mainly affect the conversion rate of biodiesel are the use of catalysts and the heating process chosen. Inductive heating is applied to improve the pyrolysis of bio-oils, a process used to obtain high quality biofuels. Inductive heating is advantageous in this process because a rapid and uniform heating of the biomass and catalyst is important for product quality. Compared to a conventional heating process, the authors found that a higher quality of bio-oil with a higher yield of aromatic hydrocarbons and a lower oxygen content is obtained in a process with RF heating [41].

**Scheme 5:**
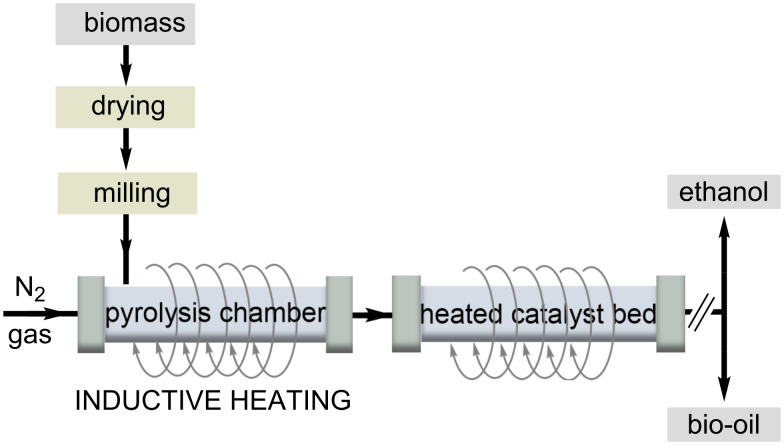
Biofuel production from biomass using inductive heating for pyrolysis.

A potential energy source for biofuel production is napier grass. The group of Lin investigated the yield and pyrolytic products of HF heating in the pyrolysis of napier grass (Scheme 5) [42]. The yield of liquid products increased with heating rates up to 150 °C/min. Pine sawdust and its major components, lignin and cellulose, were pyrolyzed by RF heating at high temperatures ranging from 500 °C to 700 °C. The authors found that higher temperature resulted in higher gas yield and lower liquid yield. The results could be relevant to the forestry and paper industries, which produce large amounts of lignin as a byproduct. The authors compared the fast pyrolysis of poplar wood and switchgrass by RF heating. The highest yield of bio-oil was obtained for switchgrass at a pyrolysis temperature as low as 450 °C.

#### 2.8 Water electrolysis

**Scheme 6:**
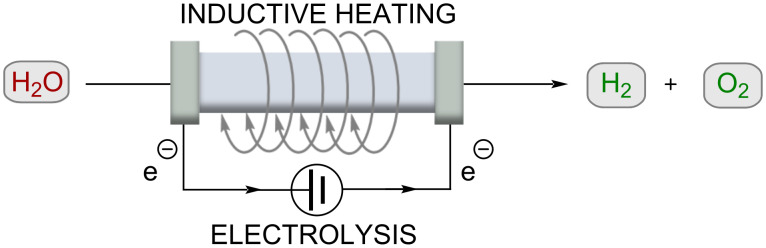
Water electrolysis using an inductively heated electrolysis cell.

Electrolysis of water as a power-to-hydrogen (PtH) concept is not new, but has become one of the most important topics being discussed today. Due to the energy of hydrogen–hydrogen bonding, water electrolysis enables chemical storage of renewable electricity. Chatenet, Carrey and co-workers showed that the electrocatalytic reaction of hydrogen formation from water can be improved by using RF heating (Scheme 6) [43]. Thus, nickel-coated iron carbide NP (FeC-Ni) was developed to drastically reduce the overpotential at 20 mA/cm^2^ (≈200 mV for OER). This kinetic enhancement corresponds to a temperature increase of 200 °C, although the actual temperature only increased by 5 °C. The authors suggested that the use of RF heating may allow water splitting near the equilibrium voltage at room temperature. Although it was expected that the magnetic field applied by inductive heating would disturb the flowing current, mainly positive effects were observed.

### 3 Micro- and mesoflow technology and indirect heating

#### 3.1 Microwave-accelerated reactions under flow conditions

**Scheme 7:**
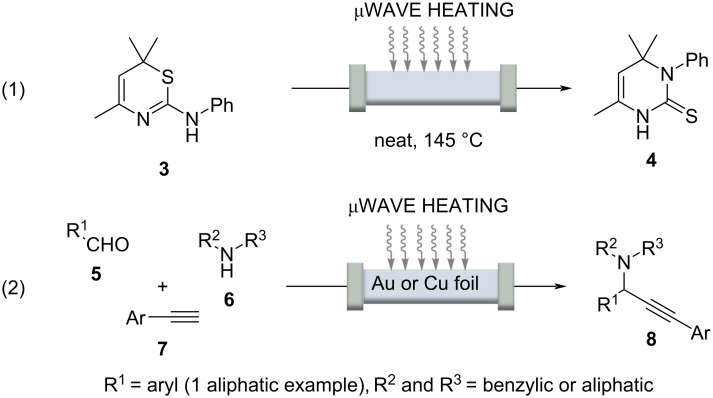
Dimroth rearrangement (reaction 1) and three-component reaction (reaction 2) to propargyl amines **8** under continuous flow conditions with microwave assistance.

Reactions that take 20 minutes or longer under classical batch conditions can be accelerated considerably under continuous flow conditions by rapid heating, because flow chemistry usually involves the use of pressure-stable reactors, which leads to shortened residence times. Inductive heating, in addition to microwave irradiation [44–46] heating, can serve as an indirect and rapid heating technology that, when combined with pressure-resistant microstructured flow reactors, enables "flash" heating so that even supercritical conditions can be achieved. In this context, Poliakoff and co-workers used supercritical water to perform several industrially relevant and continuously guided conversions, using microwave irradiation as an indirect heating method [47]. Other examples in which the two enabling technologies microwave and flow were combined are the Dimroth rearrangement exemplified for the conversion of 1,3-thiazine **3** to the corresponding 3-substituted hydropyrimidine **4** (Scheme 7, reaction 1) [48].

A noteworthy example was recently published by Organ and co-workers [49]. A three-component reaction of an aldehyde **5**, a secondary amine **6**, and a terminal alkyne **7**, afforded arylpropargylamines **8** in up to 84% yield under flow conditions (Scheme 7, reaction 2). Microwave irradiation interacted with a thin foil of Cu or Au that served as catalyst inside the glass capillary. The work must be highlighted in that the actual temperature of the glass/metal surface could be determined locally using a high-resolution IR camera. It was found to be 950 °C and not 185 °C of the reaction mixture itself. These studies are noteworthy because it can be assumed that the temperatures determined by Organ locally on metal surfaces can also be transferred to inductively heated materials including superparamagnetic nanoparticles.

#### 3.2 Reactions under flow conditions accelerated by inductive heating

**Figure 4:**
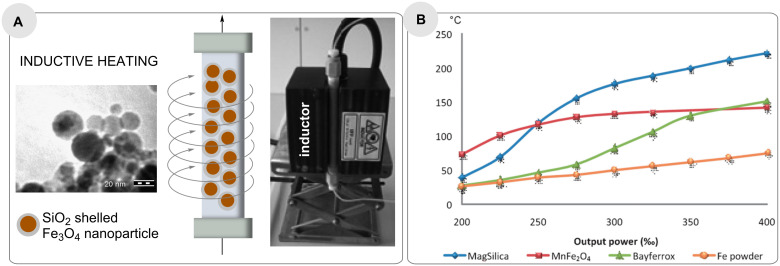
A. Flow reactor filled with magnetic nanostructured particles (MagSilica^TM^) and packed bed reactor embedded in inductor (right); B. heating profile of different materials exposed to an electromagnetic field. Heating profiles of MagSilica^TM^, MnFe_2_O_4_, Bayferrox^TM^, and iron powder.

**3.2.1 First steps and comparison with other techniques:** As mentioned in the introduction, various physical phenomena enable rapid heating of inductive materials such as steel or copper, or fixed-bed materials composed of steel beads as well as superparamagnetic nanoparticles in an oscillating electromagnetic field. Kirschning and co-workers introduced nanostructured particles based on Fe_2_O_3_/Fe_3_O_4_ coated with silicon dioxide (core-shell nanostructured particles), called MagSilica^TM^ to be used as fixed-bed materials in many different continuous flow processes (Figure 4A) [50].

These materials are excited very rapidly in a medium frequency (25 kHz) electromagnetic field, heating reaction mixtures in packed bed reactors to temperatures up to 250 °C, which was measured at the reactor outlet (Figure 4B)).

The heat is generated only at the surface of the iron oxide nanoparticles (eddy currents) and this is dissipated to the surrounding environment, which is why the bulk temperature must be much lower than the surface temperature of the nanostructured particles (Figure 4B).

**Scheme 8:**
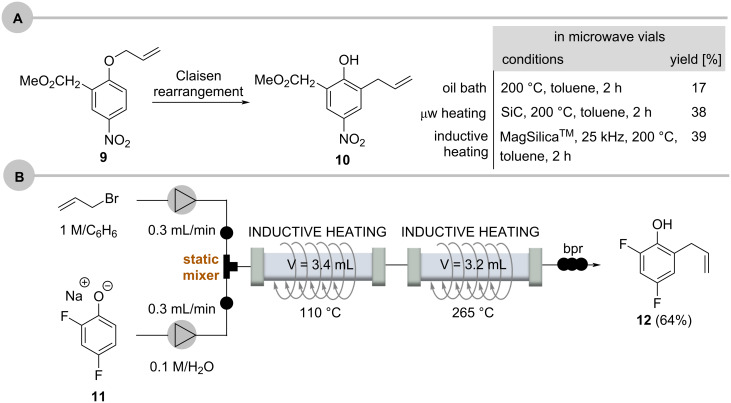
Claisen rearrangement in flow: A. comparison between conventional heating (external oil bath), microwave irradiation, and inductive heating; and B. coupled flow-through protocol consisting of *O*-allylation and Claisen rearrangement for the continuous synthesis of 2-allyl-4,6-difluorophenol (SiC = silicon carbide) [52].

The Claisen rearrangement of the electron-deficient aryl allyl ether **9** was chosen to compare the versatility and performance of inductive heating with conventional and microwave heating (Scheme 8A) [50]. The effectiveness of inductive heating is clearly comparable to microwave-induced heating. In continuation of these studies a two-step sequence was developed which showed that Claisen rearrangements can be accelerated in water as solvent (Scheme 8B) [51]. The phenolate salt **11** was mixed with allyl bromide in a static mixer and inductively heated to 110 °C at 110–160 bar to form the *O*-allyl phenol which was heated in a second reactor to 265 °C where the Claisen rearrangement under near-critical conditions occurred to yield 2-allyl-4,6-difluorophenol (**12**) in 64% yield. In this example, the two reactors made of steel were heated directly by the external electromagnetic field.

**Scheme 9:**
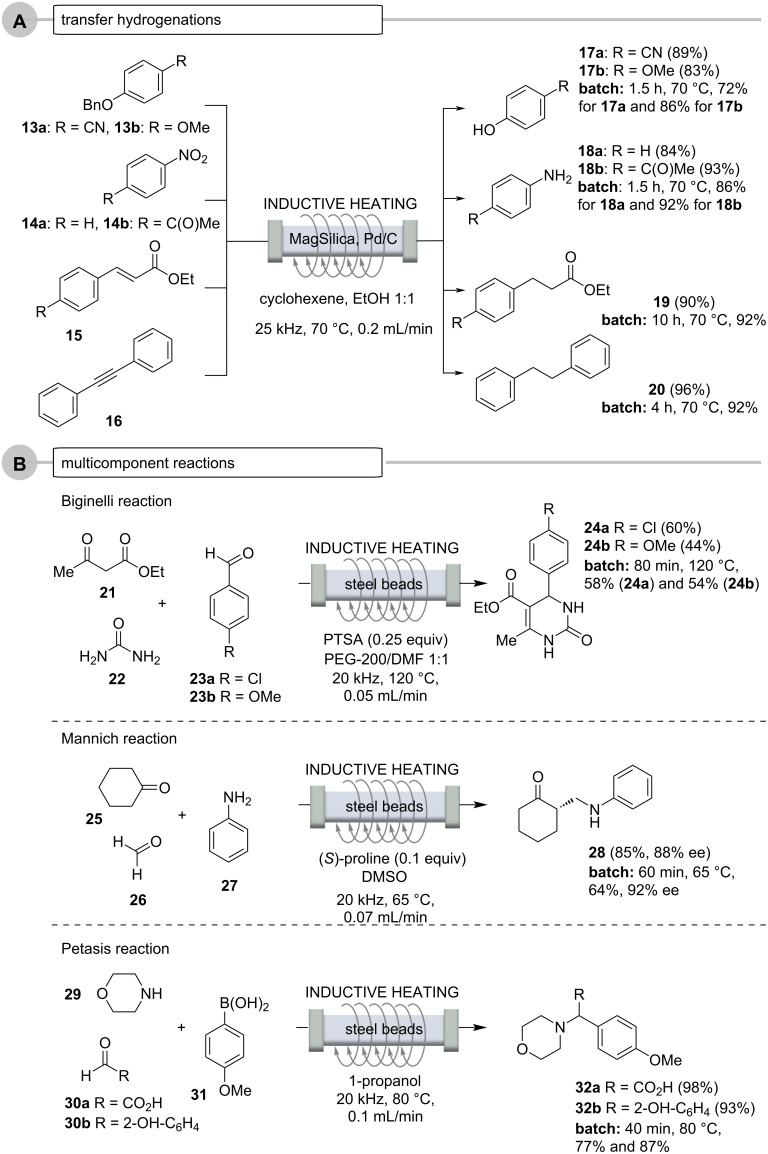
Continuous flow reactions and comparison with batch reaction (oil bath). A. Pd-catalyzed transfer hydrogenations using ethanol in cyclohexene [53], B. multicomponent reactions.

**Scheme 10:**
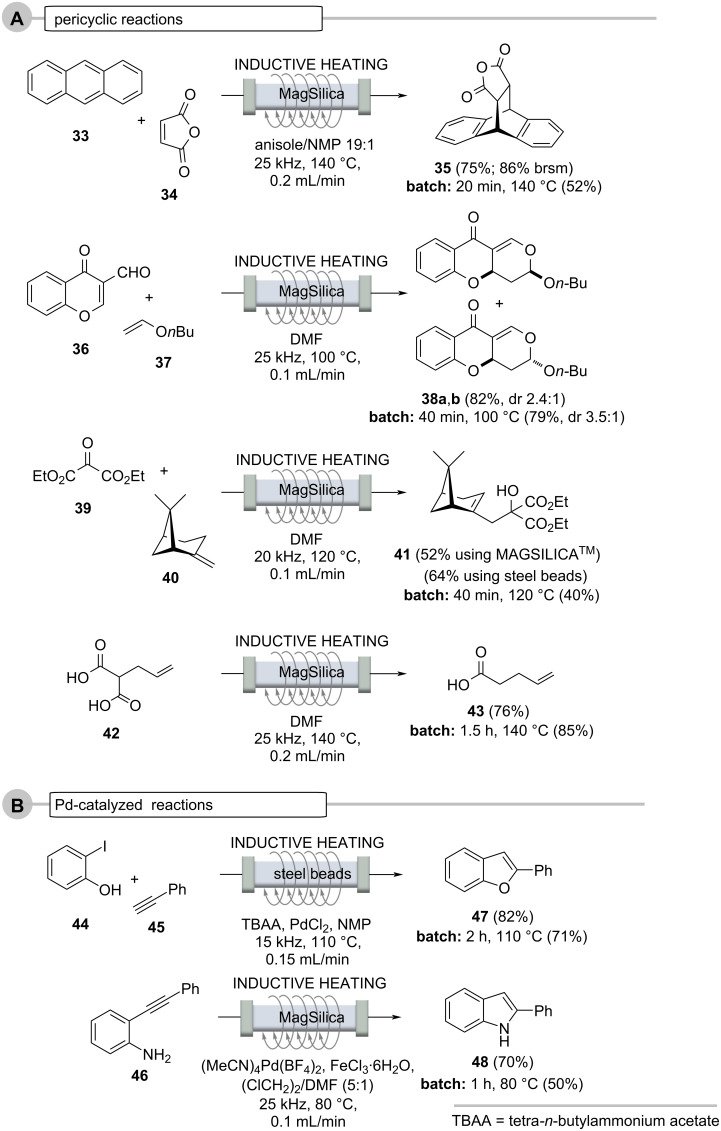
Continuous flow reactions and comparison with batch reaction (oil bath). A. pericyclic reactions and B. Pd-catalyzed reactions.

Other comparisons include the Pd-mediated transfer hydrogenations using ethanol in cyclohexene (Scheme 9, case A), multicomponent reactions (Scheme 9, case B), pericyclic reactions (Scheme 10, case A) and Pd-catalyzed reactions (Scheme 10, case B) [53]. Noteworthy, packed bed fillings used for the transfer hydrogenations (Pd) are reusable for several reductions without the need to adjust the overall reaction conditions (flow rate and residence time, temperature etc.).

Multicomponent reactions (MCRs) are of particular interest in the field of flow chemistry because this enabling technique can be easily automated. Thus, protocols can be iteratively repeated by simply changing building blocks so that compound libraries can be quickly accessed [54–55]. The formation of heterocycles traditionally often requires very harsh conditions, so that high pressure and high temperature can greatly accelerate many of these transformations. This indeed is the domain of flow chemistry. The Biginelli [56–57], Mannich [58] and Petasis [55,59–61] reactions are typical representative examples and these have been transferred into flow protocols (Scheme 9B). Steel beads serve as fix-bed materials to inductively heat up glass reactors. For the Biginelli reaction solubility of the starting urea (**22**) as well as the products **24a** and **24b** were an issue being overcome by using a solvent mixture of PEG/DMF 1:1. Also the proline-catalyzed asymmetric Mannich reaction was achieved with cyclohexanone (**25**), formaldehyde (**26**), and aniline (**27**) and 10 mol % of the organocatalyst to yield β-aminoketone **28** in 85% yield (88% ee), in less than 1 h. Although a significantly higher yield was achieved compared to the batch experiment, a slight reduction in enantioselectivity was observed. The Petasis or Petasis boron-Mannich (PBM) reaction of glyoxalic acid (**30a**) or salicylic aldehyde (**30b**), with morpholine (**29**) and *p*-methoxyphenylboronic acid (**31**) furnished α-aminocarboxylic acid **32a** and phenol **32b** in excellent yield (98% and 93%), again much higher than the yields found for the batch protocol (77% and 87%).

Pericyclic reactions such as the Diels–Alder and hetero-Diels–Alder cycloadditions, the Alder-En reaction, as well as the decarboxylation of α-alkylated malonic acids, are also suitable for flow protocols in combination with inductive heating (Scheme 10, case A) [53]. The yields but especially the residence times of the reactions outperformed those of the analogous experiments carried out under batch conditions by far [62–64].

The heating method was also successfully tested on various thermally conducted pericyclic reactions (Scheme 10, case A), such as in (hetero)-Diels–Alder reactions (anthracene (**33**) and maleic anhydride (**34**) to the cycloaddition adduct **35** and chromene carbaldehyde **36** and enol ether **37** to the diastereomeric pyrano-chromenes **38**), Alder-En reactions (oxomalonate diethyl ester (**39**) and β-pinene (**40**) to give the α-pinene derivative **41**), and the thermal decarboxylation of the malonic acid derivative **42** to give pent-4-enecarboxylic acid (**43**). In many cases, the flow protocol provided improved yields compared to the corresponding batch syntheses.

Palladium-catalyzed cross-coupling reactions require higher temperatures and thus can be realized in an inductively heated flow system [65–71]. This is exemplified for the tandem synthesis of benzofuran **47** and phenylindole **48** (Scheme 10, case B) starting from phenol **44** and aniline derivative **46**, respectively. The latter reaction was carried out in a glass reactor filled with MagSilica^TM^ [53].

**Scheme 11:**
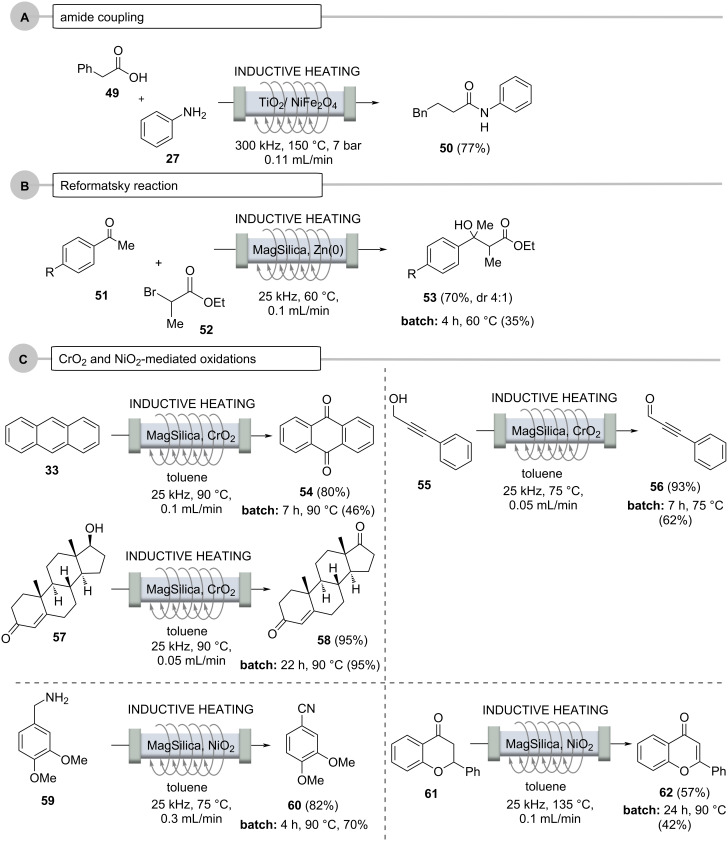
Reactions under flow conditions using inductively heated fixed-bed materials serving as stoichiometric reagents.

**3.2.2 Using chemically active fixed-beds (stoichiometric reagents):** Flow chemistry can be advantageously combined with the use of chemically active fixed-bed materials, especially heterogeneous catalysts. Here, too, the reactor material can either be heated directly by induction or there are additives in the fixed-bed material that interact with the oscillating electromagnetic field. Recently, Rebrov and co-workers disclosed the direct amide formation of a carboxylic acid and anilline using high energy ball milling to prepare the sulfated TiO_2_ (50 wt %)/NiFe_2_O_4_ (50 wt %) catalyst that serves as fixed-bed material (Scheme 11, case A) [72–73]. The reaction was carried out at 150 °C and an internal pressure of 7 bar. Remarkably, the process could be operated for 15 h with a slight decrease of effciency. Importantly, the catalyst activity can completely be restored when heating the packed bed to 400 °C exposed to an air flow. The importance of this study is the fact that no activating agents are required and water is the only byproduct.

Organometallic chemistry and heating are not among the most intuitively sensible combinations. However, prior to the formation of an organometallic species, e.g., from the metals magnesium or zinc, (thermal) activation is required. This was demonstrated for the Reformatzky reaction (Scheme 11, case B) [53,74], in which zinc powder was mixed with MagSilica^TM^ and positioned inside the flow reactor. For example, 2-bromopropanoic acid ethyl ester (**52**) and acetophenone **51** were reacted in a heated fixed-bed reactor with the mediation of zinc to give the Reformatsky product **53**, and, as commonly observed, in significantly improved yields compared to the corresponding batch processes.

Oxidations, especially metal oxide-based variants, are among the most frequently performed chemical reactions. Interesting examples are MagTrieve^TM^, which contains CrO_2_ and nickel peroxide (NiO_2_). Both were mixed with MagSilica^TM^ and used as fixed-bed materials (Scheme 11, case C) [75].

At this point, it is important to note that CrO_2_, despite its paramagnetic properties, does not heat up in an oscillating electromagnetic field because it does not exhibit conductive properties, so it had to be mixed with MagSilica^TM^. Several oxidations were performed, including those of anthracene (**33**), propargyl alcohol **55** and testosterone (**57**), which proceeded smoothly with 80%, 93%, and 95% yields, respectively, in a fraction of the time required for the corresponding batch processes. In a simplified purification protocol, potential metal impurities were then removed using a magnet. This approach could facilitate the use of metal oxides in industry for a broader range of oxidative applications. NiO_2_, on the other hand, was used to achieve the dehydrogenation of amines (to nitriles) and to perform the α,β dehydrogenation of ketones **61**.

**Scheme 12:**
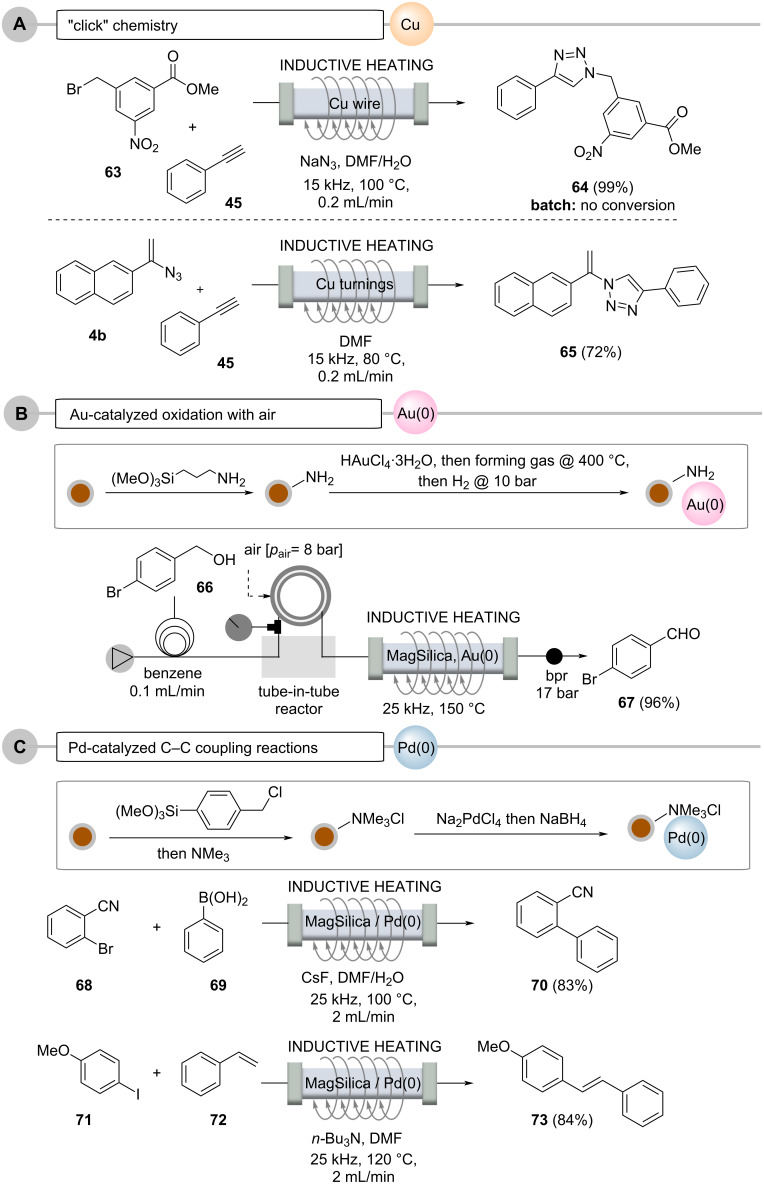
Reactions under flow conditions using inductively heated fixed-bed materials serving as catalysts: A. with copper metal, B. with Au-doped MagSilica^TM^, and C. with Pd-doped MagSilica^TM^.

**3.2.3 Using chemically active fixed beds (catalysts):** Copper metal in the form of wires or turnings can also be inductively heated when placed inside flow reactors (Scheme 12, case A). There, it performs a second role by also becoming a source for a copper catalyst, either by being released into solution or by acting as a surface-active species capable of promoting "click" reactions between alkynes and azides [76–81]. The process can be coupled with in situ generation of the azide from the corresponding bromide. The 1,2,3-triazoles are formed in up to 99% yield and in less than 10 minutes residence time, which includes azide formation prior to the cycloaddition step. Interestingly, this process could not be successfully repeated under conventional batch conditions. Organ's findings [49] suggest that the inductive heating technique creates local hot spots, either on the copper surface due to skin depth effects or alternatively in copper nanoparticles released into solution, likely leading to a dramatic acceleration of the cycloaddition reaction.

Reactions with soluble metal complexes or metal nanoparticles, utilized in transition-metal catalysis, are often avoided, especially if the metal contamination in the product exceeds certain limits. This is particularly true for the pharmaceutical industry. A continuous flow protocol for oxidations of alcohols to aldehyes or ketones using gold nanoparticles in the presence of oxygen gas or atmospheric air was achieved by modifying the silica shell of nanostructured MagSilica^TM^ with gold nanoparticles (Scheme 12, case B). After heating these modified SPIONs in an electromagnetic field, a continuous process could be established by oxidation with molecular oxygen introduced into the reaction stream via a tube-in-tube membrane reactor, a process which should be very attractive for industrial applications, as oxygen or air act as cheap and environmentally friendly oxidants [82].

An interesting combination of SPIONs and transition-metal catalysis opens up when both concepts are combined architecture-wise [83]. For instance, catalytically active metal nanoparticles, e.g., consisting of Pd(0), can be deposited on the silicate surface of MagSilica^TM^, so that the required heat for Pd(0)-mediated catalysis can be generated directly by the functionalized nanostructured particles (Scheme 12, case C) [50].

This was achieved by reductive precipitation of Pd(0) nanoparticles from ammonium-bound tetrachloropalladate [84–85], which showed good catalytic activity in various cross-coupling reactions under flow conditions. In these reactions, the leaching of palladium was as low as 34 ppm for Suzuki–Miyaura reactions and 100 ppm for Heck reactions. Importantly, the functionalized nanoparticles could be reused several times without observing a decrease in catalytic activity.

**3.2.4 Multistep processes:** The inductive heating technology has also been used in multistep processes targeting drugs or important molecules in the fragrance industry.

**Scheme 13:**
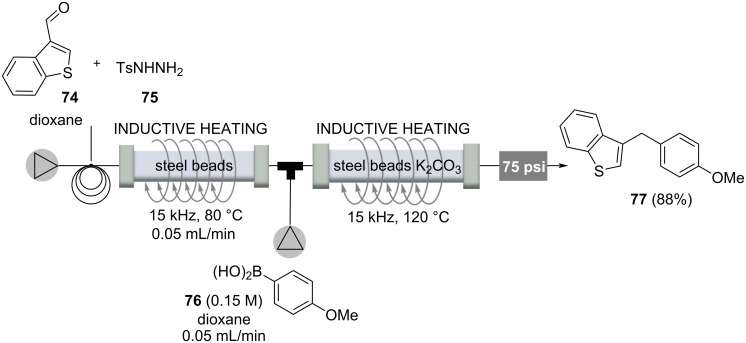
Two step flow protocol for the preparation of 1,1'-diarylalkanes **77** from ketones and aldehydes **74**, respectively, and boronic acids **76**.

The first example deals with a metal-free carbon–carbon-bond formation process between tosylhydrazones generated from the corresponding aldehydes **74** and boronic acids **76**, yielding a reduced arylation product **77** [86–87]. Mechanistically, either diazo or carbene intermediates can be proposed, as Barluenga has outlined, and migration of the aryl group leads to an alkylboronic acid, which is hydrolyzed by protodeboronation, yielding the arylation product **77**. A two-step flow protocol began with the carbonyl compounds (e.g., **74**), and the first flow step yielding tosylhydrazones that were transferred directly to the second reactor to be coupled with boronic acids (Scheme 13). Both steps required heating, which was performed by electromagnetic induction of a fixed-bed material based on steel beads. A continuous two-step flow process over a period of almost two days yielded the arylation product in 88% yield, demonstrating the robustness of the process [87].

**Scheme 14:**
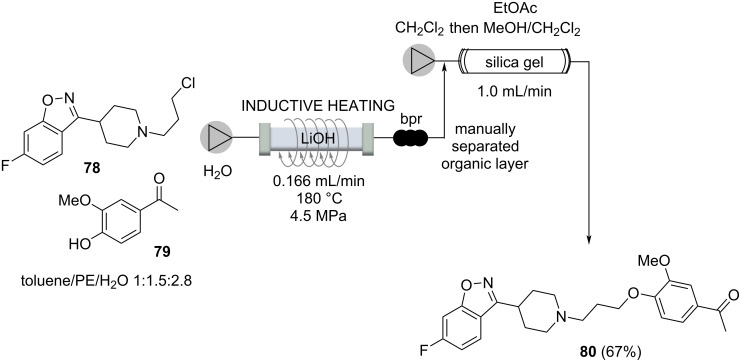
*O*-Alkylation, the last step in the multistep flow synthesis of Iloperidone (**80**) accompanied with a “catch and release” purification protocol.

The use of water as a green solvent is a greatly increasing field of research. But besides its reduced environmental footprint, water features unique physicochemical properties at supercritical conditions. The five step synthesis of a typical antipsychotic drug iloperidone (**80**) is an impressive example of how supercritical water can be utilized as a privileged solvent in organic transformations (Scheme 14) [88]. Because of space limitations, we here only highlight the last of five steps, which all involve inductive heating between 110–180 °C and four out of five steps are performed in supercritical water. Phenol **79** and the *N*-alkylated product **78** were mixed and pumped through a 1/8” stainless steel reactor, heated to 180 °C at 4.5 MPa for 7.5 min. These conditions allowed to suppress the decomposition of the *N*-alkylation product **78** by using a 1/8“-reactor. The subsequent purification was realized by a clever catch and release protocol based on a silica column, yielding iloperidone (**80**, 67%).

**Scheme 15:**
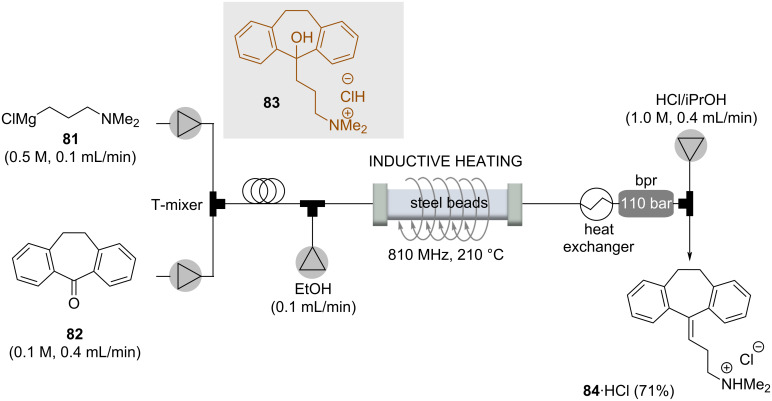
Continuous two-step flow process consisting of Grignard reaction followed by water elimination being the last steps of a multistep flow synthesis of the hydrochloride salt of amitryptiline **84**.

The tricyclic antidepressant hydrochloride of amitriptyline (**84**) was the target of a multistep continuous flow protocol in which, for one reaction step, inductive heating was used to achieve water elimination triggered exclusively under thermal conditions [54]. The flow process started with a multilithiation sequence which included a carboxylation and a Parham cyclization and hence a Grignard alkylation of ketone **82** using reagent **81**. The resulting alcohol **83** was subjected to thermolysis that led to water elimination. This step proceeded in just 30 s by employing the inductive heating technique. The crude elimination product was then mixed with a 1 M HCl solution in iPrOH that initiated crystallization of the hydrochloride salt of amitryptiline (**84**) (Scheme 15).

**Scheme 16:**
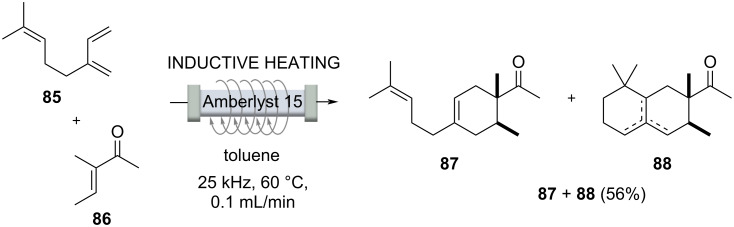
Inductively heated continuous flow protocol for the synthesis of Iso E Super (**88**) [91–92].

Iso E Super^®^ (**88**) [89] is one of the most successful synthetic fragrances ever developed [90]. It is a component of a variety of perfumes with varying ratios and is the first example of a single ingredient sold as perfume in the fragrance industry. Structurally, it is related to natural terpenes. Starting from myrcene (**85**), an inductively heated process was developed, initiated with a Diels–Alder cycloaddition that furnished ambrelux (**87**) (Scheme 16). This was cyclized under acidic conditions with Amberlyst 15^TM^ ion exchange resin embedded inside the flow reactor. However, successful conversion to an industrial process was hindered by the fact that polymerization of the starting material myrcene (**85**) could not be suppressed, leading to fouling of the catalyst and consequently to inactivation. The polymerization could be suppressed by preloading the reactor with the vinyl methyl ketone **86** before starting the process. Nevertheless, it could not be sustained over a longer period of time. By splitting the process into two independent operations, a yield of 56% (**87** + **88**) was obtained for the Diels–Alder cycloaddition, and suppressed polymerization at room temperature. This mixture was then converted in a second step and an Amberlyst 15^TM^-catalyzed cyclization at 60 °C gave **88** with a selectivity of 95%. Reactions such as polymerizations that inhibit the catalyst are side reactions that are very difficult to control, but this example sheds light on an often overlooked limitation of flow processes.

**Scheme 17:**
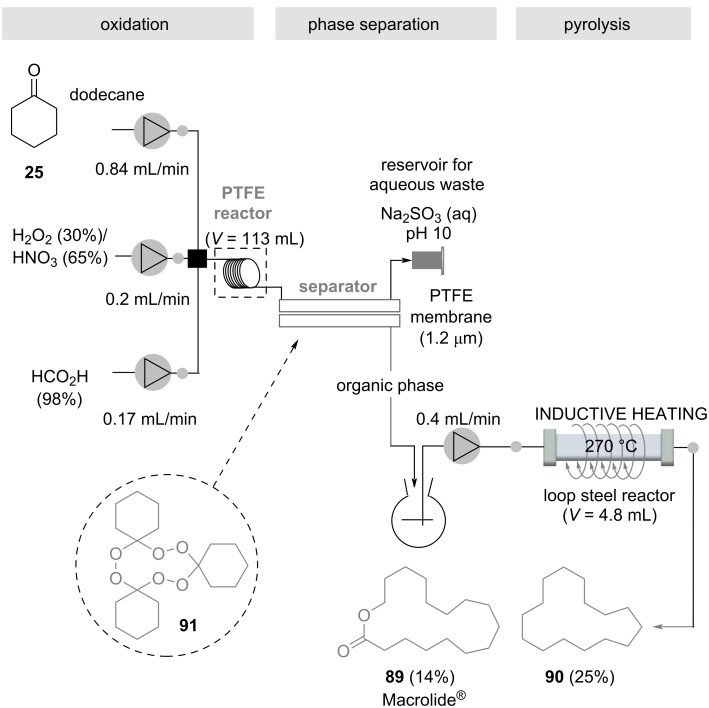
Three-step continuous flow synthesis of macrocycles **89** and **90** with musk-like olfactoric properties.

Musk-like fragrances occupy a special position among perfumes. An illustrative multistep protocol with practical relevance to the fragrance and flavor industry is a three-step flow-through protocol leading to macrocycles with musk-like olfactoric properties, which was realized under extreme conditions. These include the use of safety-hazardous reaction mixtures, the handling of explosive intermediates, and their pyrolysis at high temperatures (Scheme 17) [93]. Cyclohexanone (**25**) was mixed with conc. formic acid, and a mixture of H_2_O_2_ (30%)/HNO_3_ (65%) in a PTFE reactor at rt. This led to the formation of the cyclic triperoxide **91** in 48% (isolated) yield. Interestingly, the equilibrium favors the formation of the trimer **91** over the corresponding dimeric diperoxide. The reaction mixture was then transferred to a continuous phase separator equipped with a semipermeable membrane, from where the organic phase was transferred to a stainless steel loop reactor. Here, the macrocyclic triperoxide **91** was subjected to pyrolysis at 270 °C. This was done by inductive heating and the residence time was only 12 minutes. The Macrolide^®^ **89** was obtained in 14% together with the aliphatic macrocyclic **90**, the latter can be oxidatively converted into the corresponding ketone, which is of practical importance in the fragrance industry. It is clear that this process could not be established as a batch protocol due to the hazardous conditions.

## Conclusion

Inductive heating and flow chemistry are an ideal combination for performing continuously operated high-temperature and high-pressure syntheses. The technical setup is quite simple compared to corresponding microwave devices, the heating process is very efficient, and energetically extremely favorable. Remarkable examples in the field of fundamental chemical processes in a world that requires new solutions for energy supply show the power of inductive heating. In addition, academic examples draw attention to the use of continuously operated chemical processes with induction heating for the fields of bulk chemical production and the fragrance and flavor industries, as well as, eventually, the pharmaceutical industry. The authors are certain that this combination of enabling technologies holds great future opportunities.
